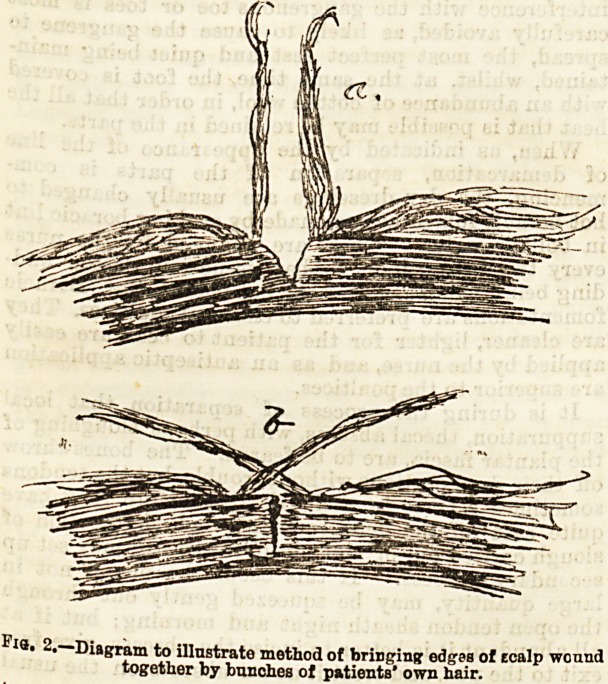# The Treatment of Injuries to the Scalp

**Published:** 1893-05-06

**Authors:** 


### ROYAL INFIRMARY, EDINBURGH.

### The Treatment of Injuries to the Scalp.

The aphorism that "no injury of the head is too slight to be despised" is as true, and, from the point of view of treatment, not less important than the other that "no one is too severe to be despaired of."

From want of careful treatment many scalp wounds, trivial in themselves, become sources of great danger to the patients, and not unfrequently assume considerable medico-legal importance. This is largely due to the fact that the skull and its contained brain and membranes, share in the damage caused by the violence which injures the scalp, and are consequently more liable to suffer from any inflammatory and septic condition resulting in the external wound.

Fig. 1.—Diagram of Scalp. *a*, Skin of scalp with blood vessels; *b b*, Occipito frontalis muscle, with aponeurosis between two bellies; *c*, Pericranium, with blood vessels passing into; *d*, bone; *e*, first space; *f*, second space; *g*, third space: *h h*, Suture, shewing pericranium bound down to bone there.

In considering the diagnosis and treatment of scalp wounds, it is essential to bear in mind the anatomical arrangements of parts. The skin of the scalp (*a*) is thick, fibrous, and dense, and is attached here and there by fibrous bands to the subjacent aponeurosis of the occipito-frontalis muscle (*b*), the intervening spaces being filled with thick granular fat.

The under surface of the occipito-frontalis is smooth, and is but loosely attached to the pericranium (*c*) by a small quantity of open areolar tissue. The pericranium is intimately connected with the bone by hosts of small blood vessels.

There are therefore three distinct spaces between the different constituent elements of the scalp: (1) between the skin and the occipito-frontalis (*e*); (2) between the occipito-frontalis and the pericranium (*f*); and (3) between the pericranium and the bone (*g*); and the importance of any lesion depends on which of these it occurs in.

I. *Bruise.*—Blood extravasated as the result of a bruise if in the first space will be well localised, and if it diffuse itself at all will do so very slowly. No active treatment is called for. The blood will slowly be absorbed and disappear. It is important to avoid a rather common error in diagnosis in this condition. The peripheral portion of the blood clot solidifies and condenses, leaving the central part fluid and soft, and to the finger the feeling is not unlike that of a depressed fracture of the skull. Firm pressure, however, enables one to reach the solid bone and a correct diagnosis.

When the blood is extravasated into the second space it diffuses itself widely, and tends rapidly to gravitate to the regions of the eyebrows, zygomatic fossæ, and back of the neck. The patient should be put to bed and kept at rest for a few days, with an ice-bag or some evaporating lotion applied to the head.

In infants, as a result of accidents during delivery, or from falls, we frequently meet with effusions of blood into the third space, constituting what is known as a cephalhaematoma. As the blood is under the pericranium it cannot trespass beyond the area of the bone over which it happens to be (*h*), and the swelling therefore assumes the shape of that bone. A free purge, and cold applied to the head are the steps taken at first. Should the blood be slow to disappear, a small quantity may be drawn off with a carefully sterilised aspirator. It is remarkable the rapidity with which a large swelling sometimes disappears after the removal of a very small quantity of fluid.

II. *Wounds of the Scalp* also derive their importance from the depth to which they penetrate. And here it may be said that to ascertain the depth of such a wound no instrument approaches in accuracy the carefully purified finger. The probe should never be employed if the finger can be introduced.

Bleeding is the only troublesome element in the

Fig. 2.—Diagram to illustrate method of bringing edges of scalp wound together by bunches of patients' own hair.

treatment of superficial scalp wounds. The vessels cannot retract and contract as they do elsewhere, and they are so closely incorporated with the dense skin that it is difficult to seize them with forceps. Pressure carefully applied will usually suffice. The hair is cut for about an inch all round, the wound is purified with one to twenty carbolic, a nail brush being used if necessary to remove particles of stone or dirt; the edges are brought together by horsehair stitches, and a dry antiseptic dressing applied. Unless pain or discharge indicate re-dressing, it need not be taken down for a week or ten days, when it will be completely healed, and the stitches may be removed. To avoid the pain of stitching, the writer has found it useful in women and children to bring the edges together by using small bunches of hair from opposite sides of the wound.

When the second space is opened into the risk is materially increased, and all antiseptic precautions must be most thoroughly attended to. It may be advisable to introduce a cat-gut drain for a day or two in severe injuries of this class. Should the pericranium be injured the condition has become a serious one, and unless amenable to treatment the patient must be warned of his danger. A sharp purge is given, the patient is put to bed on low diet, ice is applied to the head, and of course scrupulous antiseptic care is taken with regard to the dressing.

III. *Suppuration in the scalp* may follow on any of the injuries above-mentioned. All stitches must at once be removed, the pus sought for, evacuated, and efficient drainage established. A careful watch is kept for evidences of intra-cranial trouble, and should such appear appropriate means of dealing with it must not be delayed.

## Figures and Tables

**Fig. 1. f1:**
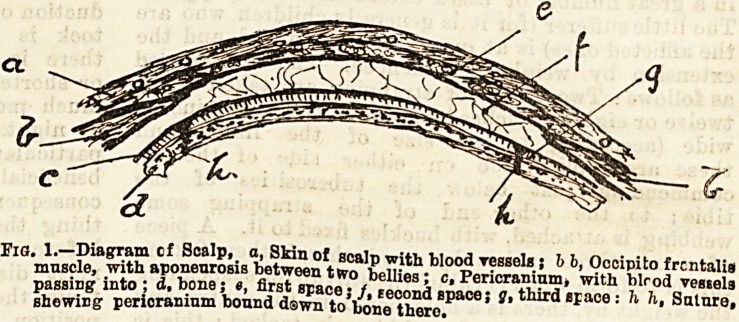


**Fig. 2. f2:**